# Curing Efficacy of Light Emitting Diodes of Dental Curing Units

**DOI:** 10.5681/joddd.2009.004

**Published:** 2009-03-16

**Authors:** Seyed Mostafa Mousavinasab, Ian Meyers

**Affiliations:** ^1^Associate Professor, Department of Restorative Dentistry and Torabinejad Research Center, School of Dentistry, Isfahan University of Medical Sciences and Khorasgan Azad University, Isfahan, Iran; ^2^Professor, Colgate Chair of General Practice Dentistry, University of Queensland, Brisbane, Australia

**Keywords:** Curing, LED, QTH lights, resin composite

## Abstract

**Background and aims:**

The aim of the present study was to compare the efficacy of quartz tungsten halogen (QTH) and light emitting diode (LED) curing lights on polymerization of resin composite.

**Materials and methods:**

A hybrid resin composite was used to prepare samples which were cured using two QTH and ten LED light curing sources. Twelve groups, each containing ten samples, were prepared using each light source. The cured depth of the resin was determined using ISO 4049 method and Vickers hardness values were determined at 1.0 mm intervals. Data was analyzed by ANOVA and Tukey test.

**Results:**

Data analysis demonstrated a significant difference between light sources for depth of cure. At 1.0 mm below the surface all the tested light sources and at 2.0-mm intervals all light sources except two (Optilux 501 and LEDemetron I) and at 3.0-mm intervals only two light sources (PenCure and LEDemetron II) could produce hardness values higher than 80% of superficial layer values.

**Conclusion:**

This study showed that a variety of LED light sources used in the present study are as effective as the high-intensity QTH lights in polymerization of resin composite.

## Introduction

Clinical performance of light-cured composite restorations is greatly influenced by the quality of curing light.^1^ Characteristics such as resin composition, light source intensity and exposure time determine the final properties of light-activated composite resins.^2^Efficacy of LED light-curing units (LCU) in polymerization of resin-based composites has been evaluated in various studies.^3-6^

According to a study performed by Obici et al^7^ there are differences between different methods of polymerization at depths greater than 2 mm, where the LED unit demonstrates the lowest depth of cure compared to QTH units.

Longer exposure time compared to QTH curing light and design improvements to increase performance in depths of cure have been proposed for first generation LED-based curing lights.^8,9^

Degree of double bond conversion of composite resin is significantly influenced by variables such as material, composite shade, depth from the surface, light source and energy level.^10-13^

Uhl et al^14^ investigated the curing efficacy of a prototype single LED light-curing unit compared to a conventional halogen one and concluded that LEDs have the potential to replace halogen LCUs if composites are selected carefully.

A number of high-power LED light sources have been marketed with a single LED to reduce curing time. To achieve this reduction in the time required for curing, these newer generations of curing lights have incorporated the latest advances in high-power LEDs so that they are capable of delivering a power density of about 1000 mW/cm^2^.^15^

Since all the spectral output of LEDs is concentrated in the blue wavelength range, more efficient curing has been shown with reduced curing time compared to the first generation LED lights and conventional halogen lamps. Thus, they would be comparable to high-intensity halogen curing lights.^16^

There are a few reports about the efficacy of newer LED curing light sources referred to as third generation LED curing lights, which deliver a broader spectral output peaking at 406 and 458 nm with a possibly better performance compared to second generation LEDs.^17-19^

This study was undertaken to assess the efficacy of some marketed LED light units including second and third generations with different light intensities and to compare them with a high-intensity and a conventional tungsten quartz halogen light with respect to depth of cure and hardness of a resin composite. We hypothesized that LED curing lights are as effective as high-intensity QTH lights in polymerization of resin composite.

## Materials and Methods

**Figure 1:**
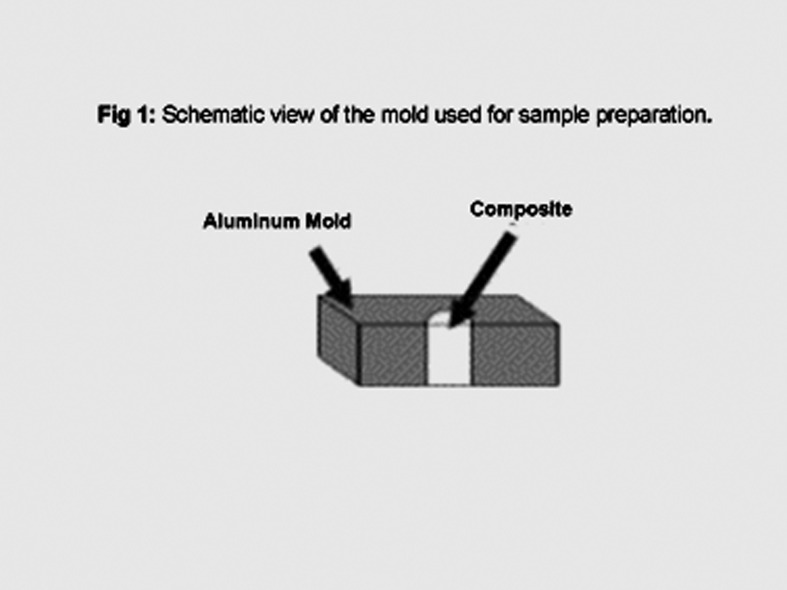
Schematic view of the mold used for sample preparation.

A hybrid resin composite (A3 shade, Filtek Z 250, 3M ESPE, St Paul, MN, USA) was used as the test material in this study. Two quartz tungsten halogen and ten light emitting diode light-curing sources used in the study are listed in Table 1. An Elipar 2500 light-curing unit was used as the control light source. A two-split aluminum mold with a semicircular, columnar hole (with a diameter of 4 mm and a depth of 8 mm) was used to prepare the samples (Figure 1). The mold was placed on a sheet of Mylar and then the resin was compressed to achieve a flat test surface, covered by a clear polyester strip (Matrix Tape Refill, 3M) and photopolymerized using light photoactivation with each of the light sources for 40 seconds in continuous mode while the light tip was in contact with the strip placed on top. Ten semicircular and column-shaped samples were prepared with each test light source (α = 0.05, power = %80, d = 0.10). The cured depth of the resin specimens was determined using International Standard ISO 4049 technique.^20^ Immediately after irradiation, uncured material was scraped away with a spatula. The height of the cylinder of set resin was measured with an electronic micrometer (Mituitoyo, Japan) to an accuracy of ± 0.01 mm, and the measured length was divided by two. Then Vickers hardness number was determined at 1.0-mm intervals along the length of the cured samples (the same samples were used for measuring the depth of cure) on the flat side perpendicular to the direction of the light source, using a universal indenter (Leitz Wetzlar; Germany) with a 100-gr load for 30 s.^21^ Data was evaluated using analysis of variance (ANOVA) and Tukey test at 95% significance level.

**Table 1:** Light sources used in the study

Light source	Manufacturer	Output (mW/cm^2^)^*^	Wavelength range (nm)^*^
Elipar 2500 Curing Light (QTH)	3M, USA	1300	400
Optilux 501 (QTH)	Kerr, USA	850-1000	380
G Light (LED)	GC, USA	>1000	465-475 (400-420)
SmartLite IQ(LED)	Dentsply Caulk,USA	1000	430
SmartLite IQ 2 (LED)	Dentsply, USA	1100	430
LEDemetron I (LED)	Kerr, USA	<1000	450
LEDemetron II (LED)	Kerr, USA	1200,1500	450
PenCure (LED)	SDI, Australia	1000	420
Elipar FreeLight 2 (LED)	3M ESPE, USA	1200	430
Satelec Mini LED (LED)	Kavo, Germany	1100	420
Radii Plus (LED)	SDI, Australia	1500	440
Radii (LED)	J Morita, Japan	1400	440

^*^ Manufacturer’s information

## Results

### Depth of cure

The results for depth of cure are shown in Table 2. Analysis of data by ANOVA showed a significant difference between the tested QTH and LED light sources for depth of cure (P < 0.001).

**Table 2:** Depth of cure according to different light sources

Groups	Means (mm) (SD)	95% Confidence Interval
Elipar 2500 Curing Light	2.77(0.12)^a^	2.67-2.85
Optilux 501	2.81(0.17)^b^	2.69-2.93
Radii	2.92(0.11)	2.84-2.99
Radii Plus	2.97(0.10)^a^	2.90-3.04
SmartLite IQ	2.90 (0.05)	2.87-2.93
SmartLite IQ 2	2.93 (0.14)	2.82-3.02
Elipar FreeLight 2	3.03(0.13)^ab^	2.93-3.12
G Light	3.10(0.16)^ab^	3.01-3.18
LEDemetron I	2.93(0.15)^b^	2.82-3.03
LEDemetron II	3.09(0.10)^ab^	3.01-3.16
PenCure	3.00(0.12)	2.91- 3.09
Satelec Mini LED	2.84(0.10)^ab^	2.92-3.08

Means with the same a and b superscripts show significant differences compared to Elipar 2500 Curing Light or Optilux 501. Means without superscripts do not show significant differences from Elipar 2500 Curing Light or Optilux 501.

Based on Tukey test results the differences between cured depth by Elipar 2500 curing light with G light, FreeLight 2, LEDemetron II, Radii Plus, and Satelec Mini LED were significant (P < 0.05).

There was a significant difference in depth of cure between Opltilux 501 and Elipar Freelight 2, G Light, LEDemetron and Satelec Mini LED light sources (P < 0.05).

No significant difference in cure depth was observed between Curing Light 2500 and Optilux 501 (P > 0.05).

### Hardness

**Fig 2:**
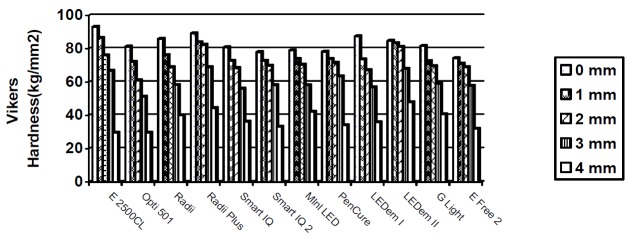
Vickers hardness values at different intervals.

The results for Vickers hardness are shown in Figure 2 and Table 3. Hardness values produced by LEDs and Optilux 501 at 0-mm, 1-mm and 2-mm intervals of the samples were significantly different from values produced by Elipar 2500 light source (P < 0.05).

**Table 3:** Mean (SD) Vickers hardness according to region and light source

Light sources	0-mm Mean (SD)	1-mm Mean (SD)	2-mm Mean (SD)	3-mm Mean (SD)	4-mm Mean (SD)	80% of surface Vikers Hardness Value
Elipar 2500 Curing Light	93.20(2.86)	86.54(1.40)	76.97(2.06)	66.85(1.68)	29.57(4.26)	74.56
Optilux 501	81.31(2.14)	72.04(2.03)	61.06(3.05)	51.30(1.62)	29.51(5.72)	65.05
Radii	85.93(1.26)	76.09(1.31)	68.94(1.09)	58.27(1.67)	40.08(1.73)	68.8
Radii Plus	89.3 (2.3)	83.9(2.9)	82.4(1.1)	68.96(1.70)	44.4(8.21)	71.47
SmartLite IQ	80.95(2.05)	72.93(1.58)	68.49(1.64)	56.12(1.09)	36.26(2.53)	64.76
SmartLite IQ 2	78.02(1.33)	72.75(0.80)	69.80(1.17)	58.20(1.85)	33.24(4.01)	62.42
Satelec Mini LED	79.06(1.65)	73.91(1.71)	70.49(1.77)	58.11(3.00)	42.21(2.01)	63.25
PenCure	78.26(1.79)	73.86(3.06)	71.61((2.79)	63.37(5.84)	34.13(2.30)	62.61
LEDemetron I	87.47(1.24)	73.6(1.02)	67.19(1.06)	56.76(1.59)	35.86(1.95)	69.97
LEDemetron II	84.80(2.37)	83.32(3.02)	81.04(0.98)	67.94(1.95)	47.84(2.79)	67.84
G Light	81.67(1.99)	72.57(1.66)	69.5(1.64)	58.91(1.14)	40.63(2.38)	65.33
Elipar FreeLight 2	74.41(0.66)	71.08(1.94)	68.97(2.00)	57.71(3.33)	32.00(2.76)	59.52

At the surface (0 mm) the highest hardness value was produced by Elipar 2500 curing light and its difference in hardness value with all the tested light sources was significant (P < 0.05).

Hardness values of surfaces polymerized by Optilux 501 and other light sources also showed significant differences (P < 0.05) except for G Light, Mini LED and SmartLite IQ.

At 1-mm interval the highest hardness value was related to Elipar 2500 curing light and its difference with all the tested lights, except for Radii Plus, was significant. The difference of the hardness produced by Optilux 501 and the other tested light sources was not significant except for LEDemetron, Radii and Radii Plus. At 2-mm interval the highest hardness value was related to Radii Plus. The differences between hardness values produced by Elipar 2500 and also by Optilux 501 compared to those gained by LEDs were significant. At 3-mm interval the highest hardness value was related to Radii Plus and all the tested LEDs except LEDemetron II, PenCure and Radii Plus compared to Elipar 2500 and all the tested LEDs compared to Optilux 501 showed meaningful differences.

At 4-mm depth the highest hardness was shown by LEDemetron II, Elipar 2500; Optilux 501 did not show significant differences compared to FreeLight 2, PenCure and SmartLite IQ 2 (P > 0.05).

All the tested light sources could produce hardness values greater than 80% of the surface hardness values of the specimens when measured 1 mm below the surface. At 2-mm interval all light sources except LEDemetron I and Optilux 501 could produce hardness values higher than 80% of the surface hardness values. PenCure and Ledemetron II were the only light sources that could cure the composite resin at 3-mm intervals so that the hardness was greater than 80% of corresponding superficial layer hardness values. At 4-mm interval the Vickers hardness values were low and well below the 80% of surface hardness values for all the tested light sources but the highest value which was about 56% of superficial layer value belonged to LEDemetron II (Table 3).

## Discussion

The aim of this study was to evaluate a variety of commercial LED curing lights, and to determine their efficacy compared to conventional quartz tungsten halogen and high-intensity quartz tungsten halogen lights. Microhardness and depth of cure were assessed since these reflect the physical properties of resin composite materials, which can be related to their clinical use. The depth of cure of a composite resin is affected by the amount of light that reaches the photoinitiator. Light intensity decreases as it passes through the sample, limiting the degree of conversion deep in the resin. Fillers and pigments strongly influence the intensity of the incident light, limiting the depth of cure. Both the intensity of the light source and attenuation of light caused by the composite resin influence the degree of conversion.^22,23^

In the present study effective hardness ratios (80% of corresponding superficial layer values) at 2-mm thickness were achieved with all the tested curing units except for LEDemetron I and Optilux 501, which is in accordance with the results of another study and can be related to differences in light intensity and energy density.^24^

Despite the presence of a significant difference in depth of cure between G Light and the QTH light sources, the G Light failed to produce a Vickers hardness value equivalent to 80% or greater than the corresponding superficial layer hardness values at 3-mm interval of the samples. In contrast, despite the absence of a significant difference in depth of cure between PenCure and QTH light sources, samples cured with PenCure could reach hardness values of 80% or greater of the corresponding superficial layer values at 3-mm intervals. A bottom-to-top hardness ratio of 80% has been reported to correspond to a bottom-to-top conversion ratio of 90%.^25^ Despite gaining the maximum hardness at 0-mm interval Elipar 2500 failed to produce hardness values greater than 80% of corresponding superficial layer hardness values at 3-mm interval.

These results indicate that the spectral distribution and intensity of curing light do not affect the depth of cure to the same degree as they do the conversion of the resin.

Based on the results of previous studies, hardness is the best predictor of monomer conversion and degree of conversion is the most sensitive test for the evaluation of the depth of cure but degree of conversion drastically reduces as the depth increases.^26,27^

LED curing lights use light emitting diodes that produce a narrow spectrum of blue light in the 400-500 nm range with a peak wavelength of about 460 nm, which is within the useful energy range for activating the photoinitiator camphorquinone (CQ) molecule, most commonly used to initiate the photopolymerization of dental monomer.^13 ,28^

The guiding principle that dictates the efficiency of a photopolymerization reaction is how much light energy is absorbed by the photoinitiator during light irradiation. Light intensity is an important factor in the activation of photoinitiator, but more importantly, it is how much of this emitted light effectively matches the absorption spectrum of the photoinitiator.^1^

Halogen curing lights showed significant differences in depth of cure and microhardness from LED curing lights. The higher intensity of Optilux can partly compensate for this difference in depth of cure, but not for hardness. Filtek Z250 resin composite uses CQ as the photoinitiator component in conjunction with a tertiary amine. Although a range of emitted light can affect the CQ absorption curve and initiate a polymerization reaction, the most efficient light absorption is at the peak maximum of 465 nm. The spectral output of halogen curing light is broad and substantial portions of the wavelengths are outside the CPQ curve. Since LED curing lights emit a narrow band of light in the range of 400-500 nm, which is in the useful energy range for activating the CPQ, the energy required to generate a given amount of radicals using LED units is smaller than that when halogen units are used.^29^

The LED light sources used in this study showed greater hardness and in most cases the depth of cure produced by tested LEDs in the present study, compared to Optilux 501, is consistent with the results of other studies.^16,30^

Some studies have shown that second generation LED LCUs are as effective as or more effective than halogen LCUs for polymerization of composite.^31,32^

PenCure and LEDemetron II were the only light sources that could cure the composite resin at 3-mm intervals so that the hardness was greater than 80% of corresponding superficial layer hardness. It is important to note that LED light curing units used in the present study are representative of high-power output LEDs and are more effective in curing the resin compared to older LED lights. Among all the tested LED light sources, PenCure and LEDemetron II had specific light output modes, which differed from other lights. In most polymerization units, light rays diverge as they exit from the output end of the light guide or emitting element; the less the divergence, the less the power density loss with increasing distance. The homogeneity of light across the existing beam is also of importance. ^2^ PenCure has a concentrated parallel light beam emitted with no light guide. The aim is to produce a uniform output with less reduction in output due to beam divergence. According to Sakaguchi and Berge^23^ maximum light intensity is achieved at 0.55 seconds, which decreases, even when set to a continuous light output method (800mW/cm^2^). It was observed that the maximum peak light emission by LEDemetron II is more adjusted to the CQ peak absorbance spectrum (468 nm) compared to other LED light sources tested in this study. LEDemetron II uses a periodic level shifting technology (PLS), which shifts the output several times within the curing time from 1500 to 1200 mW/cm^2^. Maximum light intensity is achieved every time the light pulses into the high-intensity level. This method may provide a higher amount of energy transfer to the material, which may explain higher hardness values achieved at 3-mm interval by LEDemetron II light source. However, the effect of this periodic level shifting has yet to be fully determined as it has been reported that polymerization process seems more dependent on the total energy available for photoactivation than the peak light intensity.^29^ Further research studies in this area are required.

## Conclusion

Within the limitations of this in vitro study, it can be concluded that LED curing lights are as effective as high-intensity QTH lights in polymerization of resin composite. While minor variations occurred in depth of cure and microhardness, all LED lights evaluated in this study were considered suitable for polymerization of resin composite at clinic.

## References

[1] Meniga A, Tarle Z, Ristic M, Sutalo J, Pichler G (1997). Pulsed blue laser curing of hybrid composite resins. Biomaterials.

[2] Rueggeberg FA, Caughman WF, Curtis JW JR, Davis HC (1993). Factors affecting cure depths within light activated resin composites. Am J Dent.

[3] Tsai PC, Meyers IA, Walsh LJ (2004). Depth of cure and surface microhardness of composite resin cured with blue led curing lights. Dent Mater.

[4] Bala O, Olmez A, Kalayci S (2005). Effect of led and halogen light curing on polymerization of resin based composite. Oral Rehabil.

[5] Bala O, Uçtasli MB, Tüz MA (2005). Barcoll hardness of different resin-based composites cured by halogen or light emitting diode (led). Oper Dent.

[6] Lindberg A, Emami N, Van DIJKEN JW (2005). A fourier transform raman spectroscopy analysis of the degree of conversion of a universal hybrid resin composite cured with light-emitting diode curing units. Swed Dent J.

[7] Obici AC, Sinhoreti MA, Correr SORBINHO L, Goes MF, Consani S (2004). Evaluation of depth of cure and knoop hardness in a dental composite photo-activated using different methods. Braz Dent J.

[8] Leonard DL, Charlton DG, Roberts HW, Cohen ME (2002). Polymerization efficiency of led curing lights. J Esthet Restor Dent.

[9] Bennett AW, Watts DC (2004). Performance of two blue light emitting diode dental light curing units with distance and irradiation time. Dent Mater.

[10] Yoon TH, Lee YK, Lim BS, Kim CW (2002). Degree of polymerization of resin composite by different light sources. J Oral Rehabil.

[11] Soh MS, Yap AU, Siow KS (2003). The effectiveness of cure of led and halogen curing lights at varying cavity depth. Oper Dent.

[12] Lindberg A, Peutzfeldt A, Van DIJKEN (2005). Effect of power density of curing unit, exposure duration, and light guide distance on composite depth of cure. Clin Oral Investig.

[13] Mills RW, Uhl A, Jandt KD (2002). Optical power outputs, spectra and dental composite depths of cure, obtained with blue light emitting diode (led) and halogen curing units (lcus). Br Dent J.

[14] Uhl A, Sigusch BW, Jandt KD (2004). Second generation leds for the polymerization of oral biomaterials. Dent Mater.

[15] Owens BM, Rodriguez KH (2007). Radiometric and spectrophotometric analysis of third generation light-emitting diode (led) light-curing units. J Contemp Dent Pract.

[16] Wiggins KM, Hartung M, Althoff O, Wastian C, Mitra SB (2004). Curing performance of a new generation light emitting diode dental curing unit. J Am Dent Assoc.

[17] Price RB, Felix CA, Andreou P (2006). Third-generation vs a second-generation led curing light: effect on knoop microhardness. Compend Contin Educ Dent.

[18] Owens BM, Rodriguez KH (2007). Radiometric and spectrophotometric analysis of third generation light-emitting diode (led) light-curing units. J Contemp Dent Pract.

[19] Price RB, Felix CA, Andreou P (2005). Evaluation of a dual peak third generation led curing light. Compend Contin Educ Dent.

[20] International Organization for Standardization. Dentistry - Polymer-based filling, restorative and luting materials. ISO 4049: 2000, Geneve.

[21] Koupis NS, Martens LC, Verbeeck RM (2006). Relative curing degree of polyacid-modified and conventional resin composites determined by surface knoop hardness. Dent Mater.

[22] Mccabe JF, Carrick TE (1989). Output from visible light activation units and depth of cure of light activated composites. J Dent Res.

[23] Vandewalle KS, Ferracane JL, Hilton TJ, Erikson RL, Sakaguchi RL (2004). Effect of energy density on properties and marginal integrity of posterior resin composite restorations. Dent Mater.

[24] Yazici AR, Kugel G, Gül G (2007). The knoop hardness of a composite resin polymerized with different curing lights and different modes. J Contemp Dent Pract.

[25] Bouschlicher MR, Rueggeberg FA, Wilson BM (2004). Correlation of bottom to top surface microhardness and conversion ratio for a variety of resin composite composition. Oper Dent.

[26] Dewald JP, Ferracane JL (1987). A comparison of four modes of evaluation depth of cure of conversion in a light cured composite. J Dent Res.

[27] Rueggeberg FA, Craig RG (1988). Correlation of parameters used to estimate monomer. J Dent Res.

[28] Cook WD (1982). Spectral distributions of dental photopolymerization sources. J Dent Res.

[29] Teshima W, Nomura Y, Tanaka N, Urabe H, Okazaki M, Nahara Y (2003). Esr study of camphorquinone/amine photoinitiator systems using blue light-emitting diodes. Biomaterials.

[30] Mills RW, Jandt KD, Ashworth SH (1999). Dental composite depth of cure with halogen and blue light emitting diode technology. Br Dent J.

[31] Campregher UB, Samuel SM, Fortes CB, Medina AD, Collares FM, Ogliari FA (2007). Effectiveness of second-generation light-emitting diode (led) light curing units. J Contemp Dent Pract.

[32] De ARAúJO CS, Schein MT, Zanchi CH, Rodrigues SA JR, Demarco FF (2008). Composite resin microhardness: the influence of light curing method, composite shade, and depth of cure. J Contemp Dent Pract.

[33] Sakaguchi RL, Berge HX (1998). Reduced light energy density decrease post-gel contraction while maintaining degree of conversion in composite. J Dent.

